# Efficacy and safety of thalidomide with hydroxyurea in sickle cell anemia: a quasi-experimental clinical trial

**DOI:** 10.1007/s44313-025-00068-4

**Published:** 2025-04-01

**Authors:** Priyanka Samal, Anindita Paul, Harshwardhan Bahirat, Ajit Kumar Bishoyi, Venkatarao Epari

**Affiliations:** 1 Department of Clinical Hematology, Institute of Medical Sciences & Sum Hospital, Siksha ‘O’ Anusandhan Deemed to Be University, Bhubaneswar, Odisha 751003 India; 2 Department of Community Medicine, Institute of Medical Sciences & Sum Hospital, Siksha ‘O’ Anusandhan Deemed to Be University, Bhubaneswar, Odisha 751003 India

**Keywords:** Sickle cell anemia, Thalidomide, Hydroxyurea, Clinical trial

## Abstract

**Background:**

The clinical course of sickle cell anemia (SCA) is variable, with chronic hemolysis and end-organ damage caused by microvascular occlusion. We evaluated the efficacy and safety of thalidomide plus hydroxyurea (HU) compared with HU alone to determine whether the combination provides a superior clinical benefit and safety profile.

**Methods:**

This was an open-label quasi-experimental clinical trial (Clinical Trials Registry of India, CTRI Registration Number 2023/04/065682). Patients with SCA aged > 12 years and postmenopausal females aged > 45 years were allocated 1:1 to receive either HU (20 mg/kg/day) and thalidomide (50 mg/day) in Group A or HU (20 mg/kg/day) only in Group B.

**Results:**

The frequency of vaso-occlusive crises (VOCs), transfusion requirements, variations in hematological parameters (hemoglobin [Hb], fetal hemoglobin [HbF], and sickle hemoglobin [HbS]), and side effects between the groups were assessed over 12 months. Repeated-measures analysis of variance was used to determine changes across the observation period. The mean age of the 66 patients diagnosed with SCA (homozygous HbS mutation) was 32.9 (standard deviation ± 11.5) years, and 57.6% were males. Over the 12-month observation period, Group A had significantly fewer VOCs (3.48 ± 2.81) and packed red blood cell transfusions (3.61 ± 2.19) than Group B (11.36 ± 4.20 VOCs; 13.27 ± 3.70 transfusions) (*p* = 0.0001). There was a significant increase in Hb (8.2 ± 1.8 to 11.8 ± 1.2 g/dL), a decrease in HbS% (72.5 ± 5.5 to 64.5 ± 5.4), and a rise in HbF% (18.9 ± 5.1 to 28.4 ± 5.6) (*p* < 0.0001) in Group A.

**Conclusion:**

Combining thalidomide with HU significantly reduced VOCs and transfusion requirements, improved Hb and HbF%, and decreased HbS levels.

## Introduction

Sickle cell disease (SCD) is a hematological disorder caused by a single-gene mutation in which valine replaces glutamic acid at the sixth position of the β-globin chain, leading to sickle hemoglobin (HbS) formation. Under low-oxygen conditions, red blood cells (RBCs) become rigid and sickle-shaped and obstruct the microvasculature, resulting in vaso-occlusive crises (VOCs) and increased cellular adhesiveness. The clinical course of SCD varies significantly, with complications, including chronic hemolysis and end-organ damage affecting the lungs, kidneys, liver, and brain. Homozygosity for the HbS mutation (HbSS) causes sickle cell anemia (SCA), the most severe form of SCD, while compound heterozygous states (HbSC, HbSE, HbSβ⁰) can produce similar symptoms [1]. SCA occurs when an individual inherits two copies of the mutated β-globin gene (HBB), one from each parent, leading to the exclusive production of HbS instead of normal hemoglobin (Hb) A. The resulting sickle-shaped RBCs cause vaso-occlusion, hemolysis, and various complications associated with SCD.

Globally, SCD has a high incidence, particularly in sub-Saharan Africa, where prevalence ranges from 1 to 2% [2]. India, the second most affected country, reported an estimated 42,016 SCA births in 2010, reflecting a rising prevalence of up to 4.3% in various populations, including indigenous tribes [3].

Available treatment options for HbSS include disease-modifying drugs targeting several pathophysiological pathways and definitive therapies, such as gene therapy and hematopoietic stem cell transplantation [4]. Hydroxyurea (HU), a fetal hemoglobin (HbF) inducer, has been a cornerstone of SCD management since its Food and Drug Administration approval in 1998, demonstrating its efficacy in reducing pain episodes, acute chest syndrome, and mortality [5, 6]. Recently, novel therapeutic agents, such as voxelotor, L-glutamine, and crizanlizumab, have expanded the treatment landscape by targeting different aspects of SCA pathobiology [7].

Thalidomide and its analogs are emerging as promising agents for treating HbSS because of their diverse mechanisms of action, including induction of HbF, anti-inflammatory properties, and immunomodulatory effects. It inhibits tumor necrosis factor-alpha and activates the p38 mitogen-activated protein kinase pathway, stimulating HbF production [8, 9].

We evaluated the efficacy and safety of thalidomide in combination with HU compared with HU alone in patients with SCA. The frequency of VOCs, need for transfusions, variations in hematological parameters, and side effects between treatment groups were assessed to determine whether combination therapy provides superior clinical benefit and safety profile in managing SCA (HbSS).

## Materials and methods

An open-label quasi-experimental clinical trial was conducted at the Department of Hematology and Hemato-Oncology between January 2022 and May 2023. Male patients of 12 years and above and postmenopausal females of 45 years and older were enrolled in the trial. Hemoglobin electrophoresis (MINICAP SEBIA FLEX-PIERCING capillary electrophoresis system) was performed to confirm the diagnosis of SCA. The study included two groups of participants: Group A received HU (20 mg/kg/day), thalidomide (50 mg/day), and folic acid (5 mg/day), while Group B received HU (20 mg/kg/day) and folic acid (5 mg/day). The exclusion criteria included females of childbearing age (15–45 years); significant liver, renal, cardiac, pulmonary, or neurological conditions; a history of thrombotic episodes; active human immunodeficiency virus infection; or hepatitis B or C infections.

All patients were administered a fixed low dose of HU at 20 mg/kg/day, along with supportive care. Since HU is available in India only in a 500 mg capsule formulation, the daily dosage was adjusted as necessary to maintain an equivalent dose of 20 mg/kg/day for each patient. The patients were also advised to consume folic acid (5 mg/day) and ensure adequate hydration.

The clinical responses and laboratory parameters were monitored from the initiation of HU therapy to each follow-up visit over 16 months. Monthly assessments were performed using a standardized clinical protocol. Laboratory evaluations at baseline and follow-up included Hb, mean corpuscular volume (MCV), HbF, reticulocyte count, and serum creatinine. Additional data collected included adverse effects, consanguinity, patient age, and sex. The frequency of VOCs and blood transfusions in the previous year was compared with post-treatment values. Baseline clinical and laboratory parameters (Hb, HbS, HbF, and MCV) were analyzed after 16 months of low-fixed-dose HU therapy. Patient adherence was assessed by counting the remaining HU capsules during follow-up visits.

**Fig. 1:**
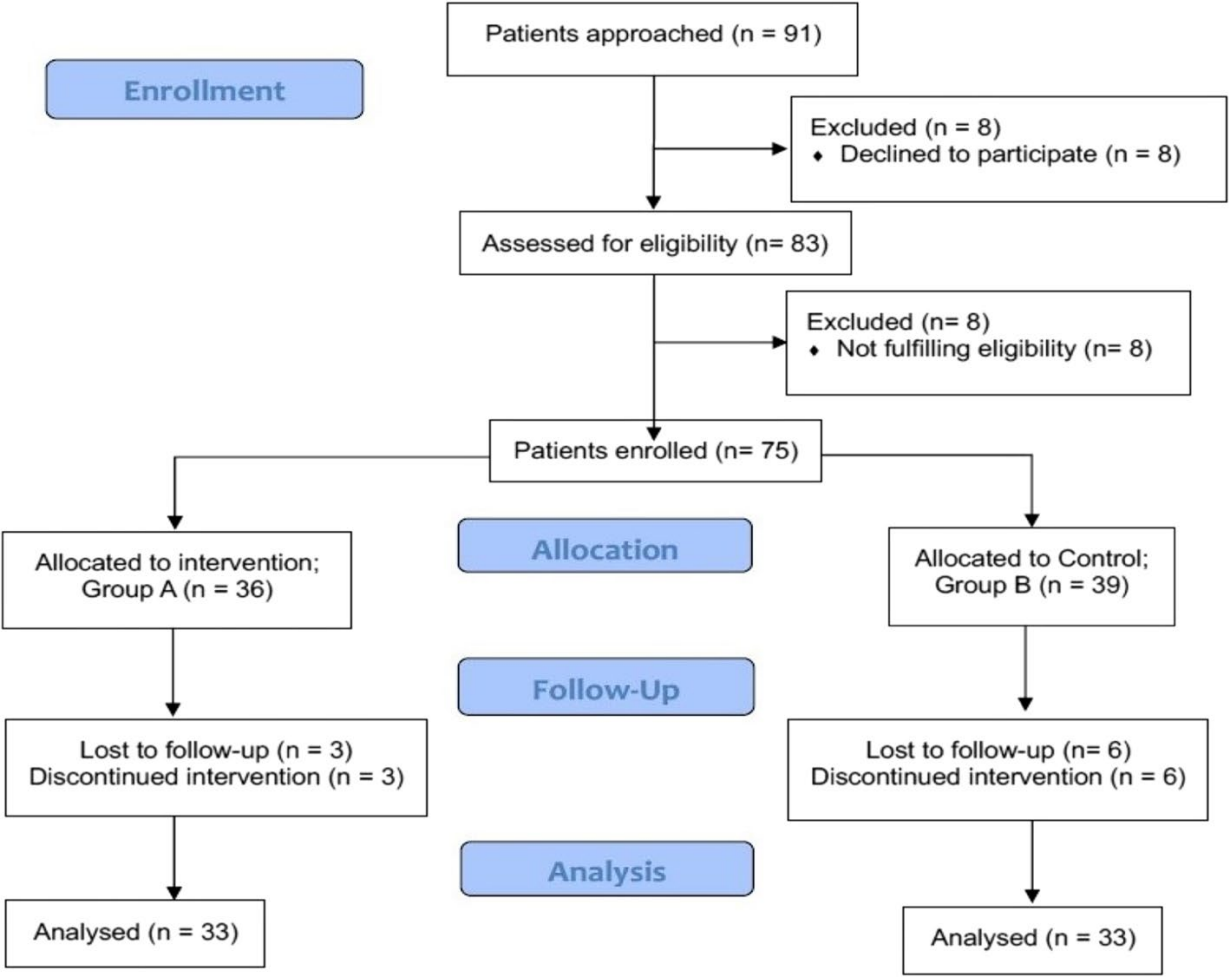
Consort flow diagram of participant enrolment and progression

As per the enrollment, allocation, follow-up, and analysis of participants in the clinical trial, 91 patients were initially approached, of whom 8 declined to participate, leaving 83 assessed for eligibility. Among them, 8 were excluded because they did not meet the eligibility criteria, resulting in 75 enrolled patients. These participants were randomly assigned to two groups: Group A (n = 36) received the intervention, and Group B (n = 39) served as the control group. During the follow-up period, 3 patients from Group A and 6 from Group B were lost to follow-up, leading to the final analyzed samples of 33 patients in each group (Fig. 1).

Based on the order in which they were presented, patients were enrolled in either group based on their willingness to participate. This study did not employ blinding or treatment concealment. Using the G Power 3.1.9.4 program, the sample size calculation indicated that 66 patients, or at least 30 in each group, were required to attain adequate statistical power.

At the start of the trial, each participant underwent comprehensive clinical and laboratory evaluations, followed by periodic follow-up assessments at 3, 6, 9, and 12 months. Parameters, including hemoglobin electrophoresis, renal and liver function tests, complete blood counts, frequency of VOCs, need for blood transfusions, and safety profile, were monitored and compared over time.

Pain intensity was assessed using the Numeric Rating Scale, while adverse events were monitored following the Common Terminology Criteria for Adverse Events Version 5.0 [10, 11]. The five primary outcome variables were Hb increment, reduction in VOCs, decreased transfusion requirement, decreased HbS%, and increased HbF%. Transfusion independence, defined as a reduction in the need for transfusion, was used as the response criterion. Patients were categorized as major responders if their hemoglobin increased by ≥ 2 g/dL, minor responders if it increased by 1–2 g/dL, and non-responders if there was no significant change [12].

The choice of a quasi-experimental study design was driven by several practical and ethical considerations. First, we included patients with SCA (HbSS), a vulnerable population with significant disease-related challenges. Randomization was deemed impractical as it could limit patient autonomy and adherence to treatment regimens. Instead, the participants were enrolled in treatment groups based on their willingness, reflecting real-world clinical scenarios and optimizing compliance. Second, distinct therapeutic agents (HU and thalidomide) made blinding and treatment concealment infeasible, reinforcing the suitability of a quasi-experimental design. Additionally, recruiting a sufficient number of participants for a fully randomized controlled trial in a single-center setting posed logistical constraints, particularly given the rarity of SCA (HbSS) and its complex clinical management. This approach allowed us to evaluate the comparative effectiveness of the interventions in routine clinical practice while ensuring ethical standards, feasibility, and resource optimization. These factors collectively justified the adoption of a quasi-experimental design for this investigation. Ethical clearance for the study was obtained from the institutional ethics committee (ref. no. IEC/IMS.SH/SOA/2021/270 dated December 27, 2021).

IBM SPSS Statistical Software v27.0 was employed for the data analysis. Continuous variables are expressed as mean and standard deviation. Frequency distribution and Chi-square tests were applied to analyze categorical variables, and paired/independent sample t-tests were used to evaluate continuous variables. Repeated-measures analysis of variance was used to assess the changes across the observation period of 12 months, with the F-value calculated using Wilks’ Lambda test. Mauchly’s Test of Sphericity was used to test the assumption of sphericity, and when violated, a Greenhouse–Geisser correction was used. A *p*-value of < 0.05 was used to signify statistical significance.

## Results

This study included 66 patients with SCA (HbSS). Figure 1 shows a schematic representation of the patient enrollment. The average age was 32.9 ± 11.5 years. Most (56.1%) patients were in the 21–40 age group, with males constituting 57.6%. Table 1 shows the baseline characteristics of the study participants, including their clinical and biochemical parameters. The baseline packed red blood cell (PRBC) requirement/year (5.27 vs. 5.03) and VOC episodes/year (11.18 vs. 10.79) between the groups before enrollment were statistically comparable (*p* = 0.467 and 0.501, respectively), although hemoglobin, percentage of reticulocytes, and HbF, among other hematological parameters, were not comparable.

**Table 1:** Baseline demographic characteristics, clinical and biochemical parameters of the study participants (*n* = 66)

**Variables**	**Group A** Thalidomide + Hydroxyurea + Folic acid n (%)	**Group B** Hydroxyurea + Folic acid n (%)	***p*-value**
Age group	4 (12.1)	6(18.2)	0.375
≤ 20			
21—40	17(51.5)	20(60.6)	
> 40	12(36.4)	7(21.2)	
Sex	21(63.6)	17(51.5)	0.228
Male			
Female	12(36.4)	16(48.5)	
Mean Hb (gm/dl)	8.2 ± 1.8	9.4 ± 1.1	0.002
Reticulocyte count (%)	3.29 ± 1.26	5.36 ± 2.26	0.001
MCV	85.33 ± 3.73	82.9 ± 9.18	0.167
HbS %	72.5 ± 5.5	73.3 ± 7.7	0.616
HbF %	18.9 ± 5.1	15.6 ± 6.7	0.027
PRBC Transfusions	5.3 ± 1.0	5.0 ± 1.6	0.467
VOCs	11.2 ± 2.4	10.8 ± 2.3	0.501
Serum Creatinine	2.37 ± 1.03	3.09 ± 1.68	0.043
Total Bilirubin	3.01 ± 1.76	5.05 ± 1.56	0.001
Direct Bilirubin	1.16 ± 1.12	0.68 ± 0.72	0.045

**Fig. 2:**
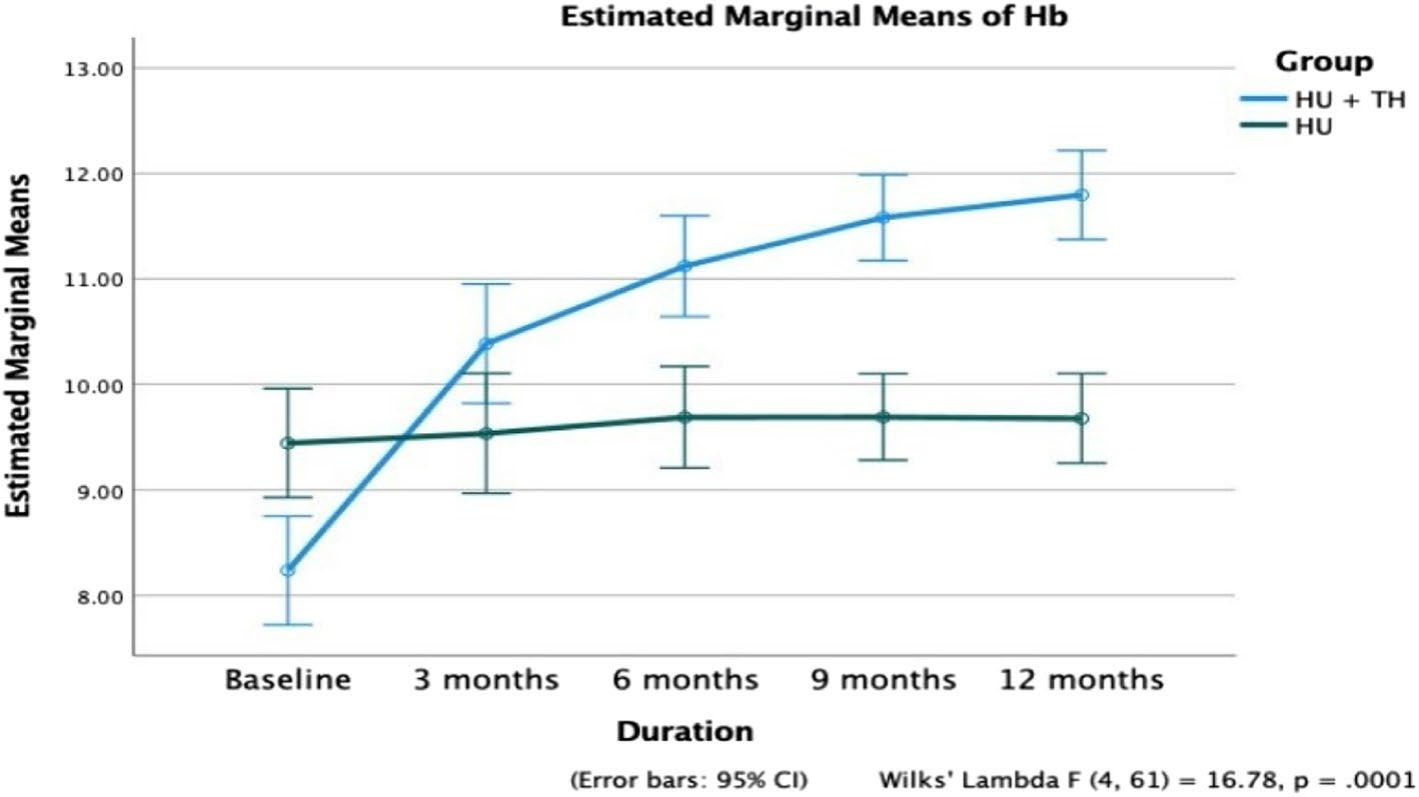
Comparison of hemoglobin (Hb) levels over 12 months in Groups A and B

**Fig. 3:**
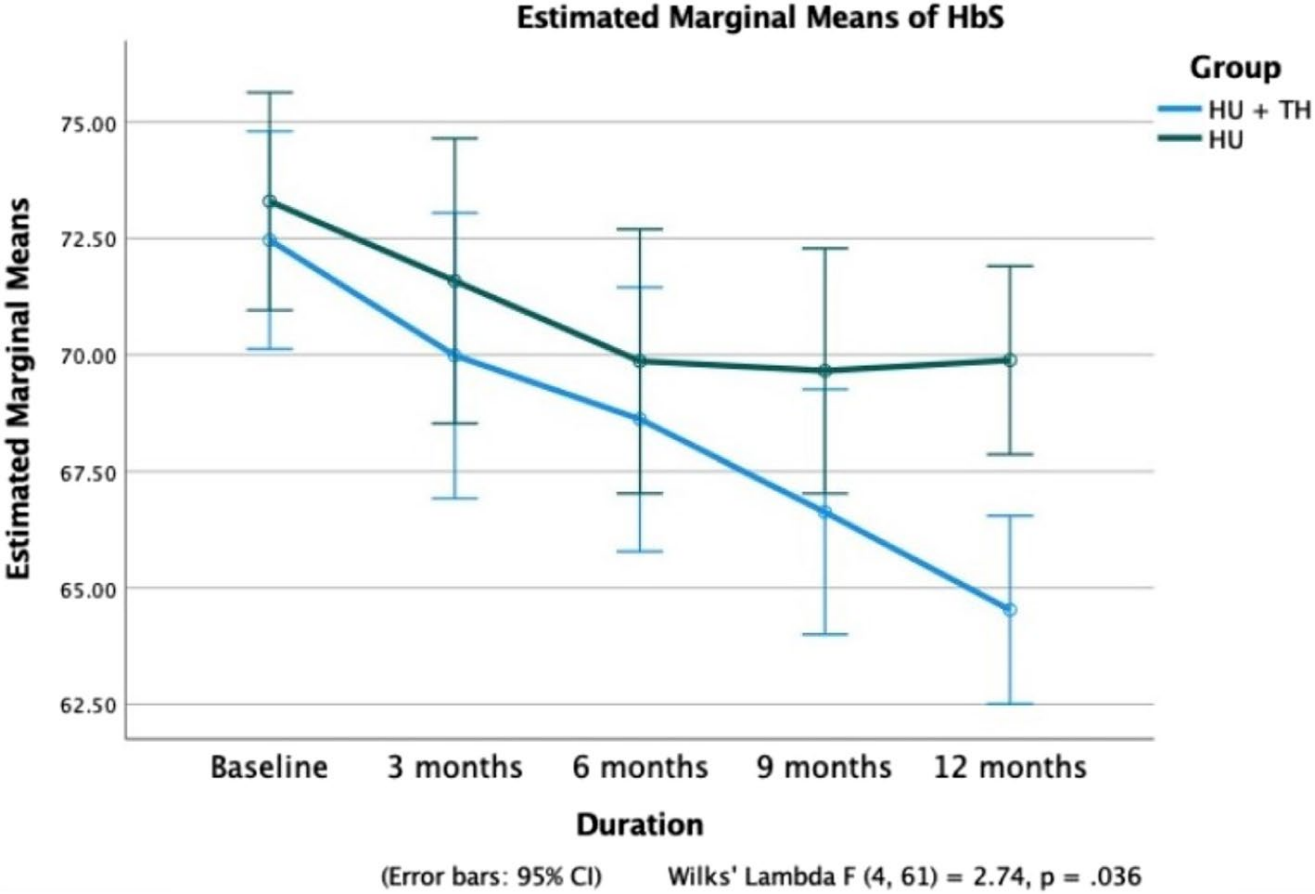
Comparison of hemoglobin S (HbS) percentage over 12 months in Groups A and B

Figures 2 and 3 show comparisons between the two intervention arms. The 12-month treatment regimen with thalidomide in Group A patients had a statistically significant effect on all five outcome measures compared with their counterparts in Group B.

**Fig. 4:**
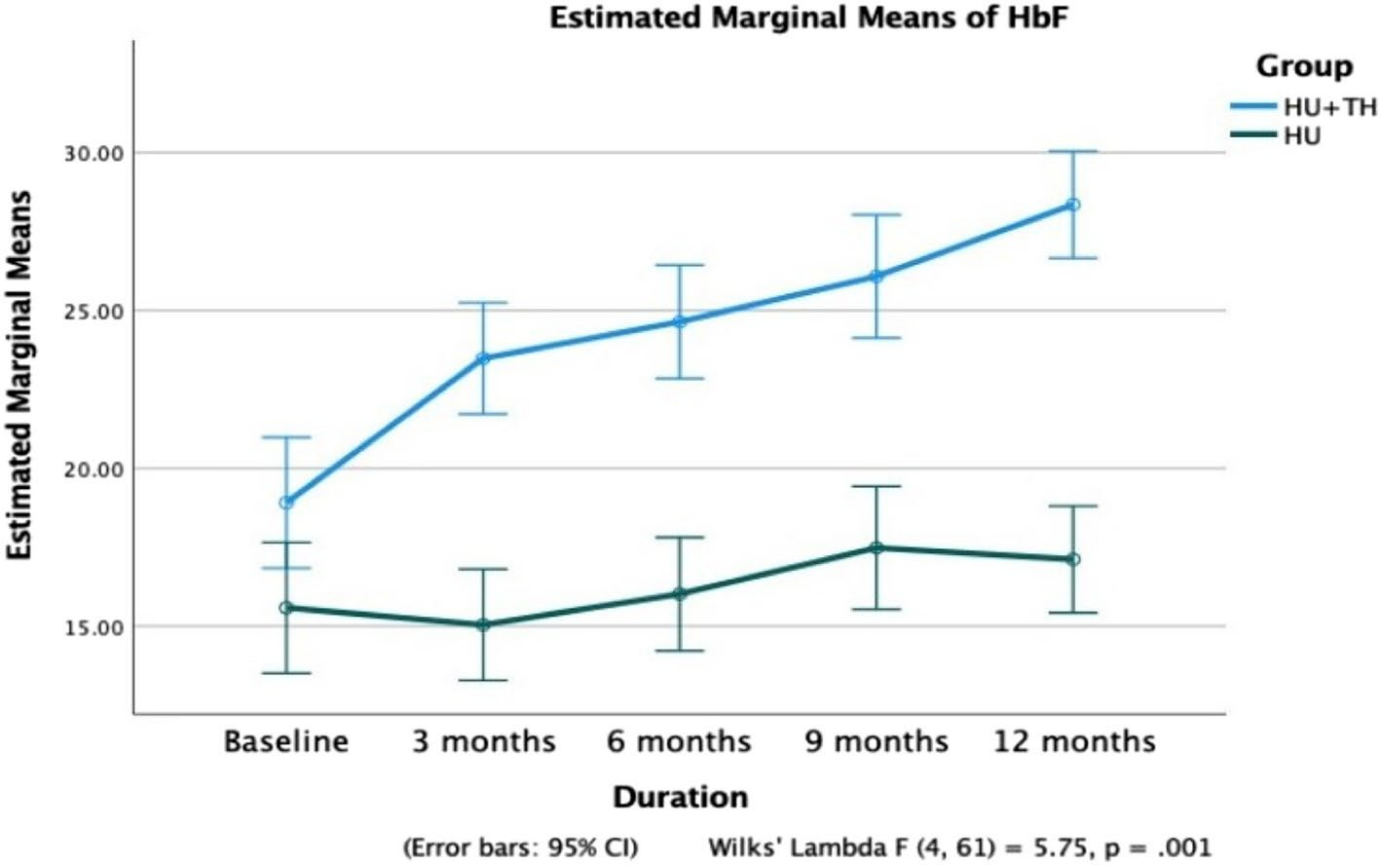
Comparison of fetal hemoglobin (HbF) between Groups A and B over a 12-month period

**Fig. 5:**
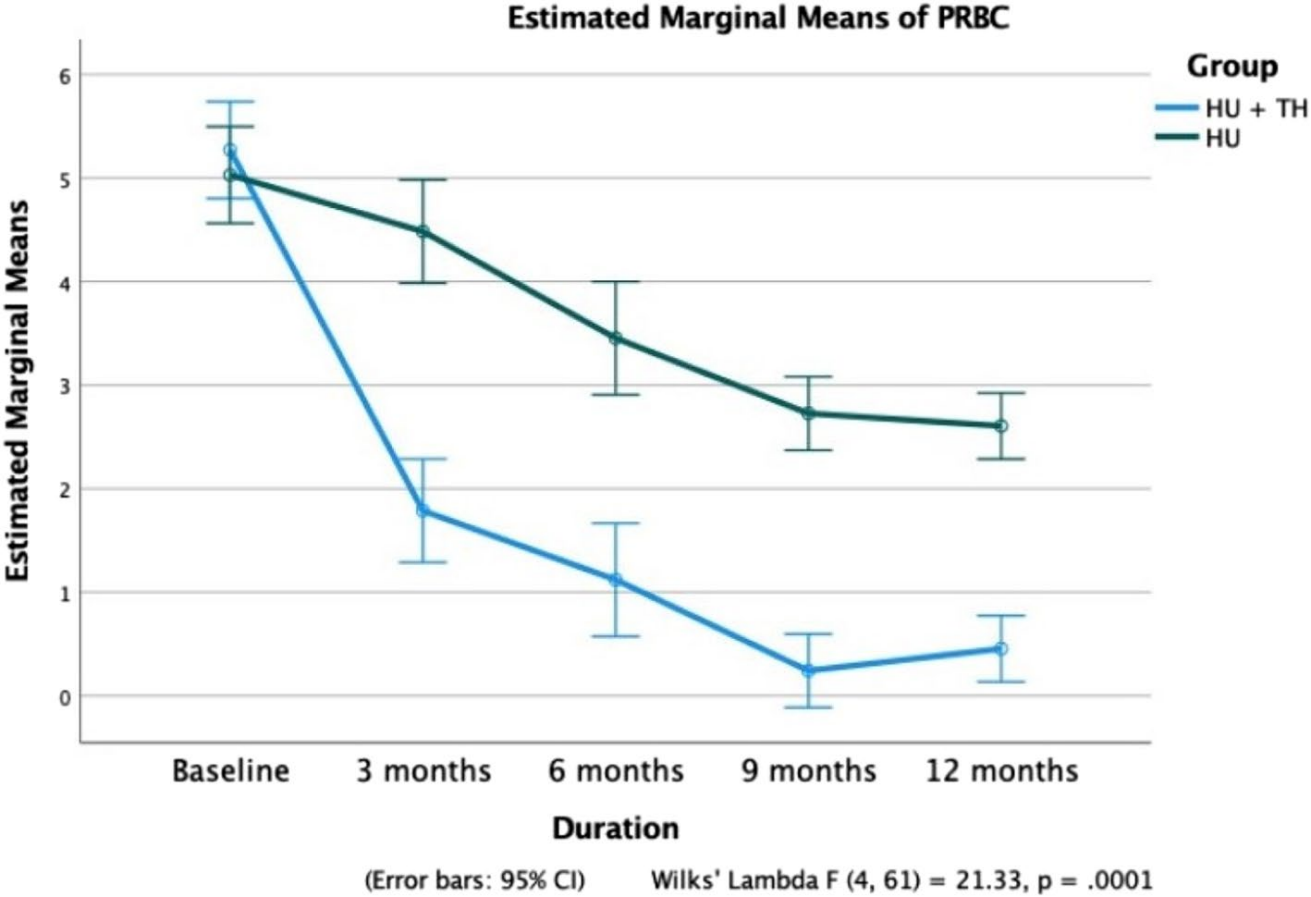
Comparison of mean packed red blood cell (PRBC) transfusions between Groups A and B over a 12-month period

**Fig. 6:**
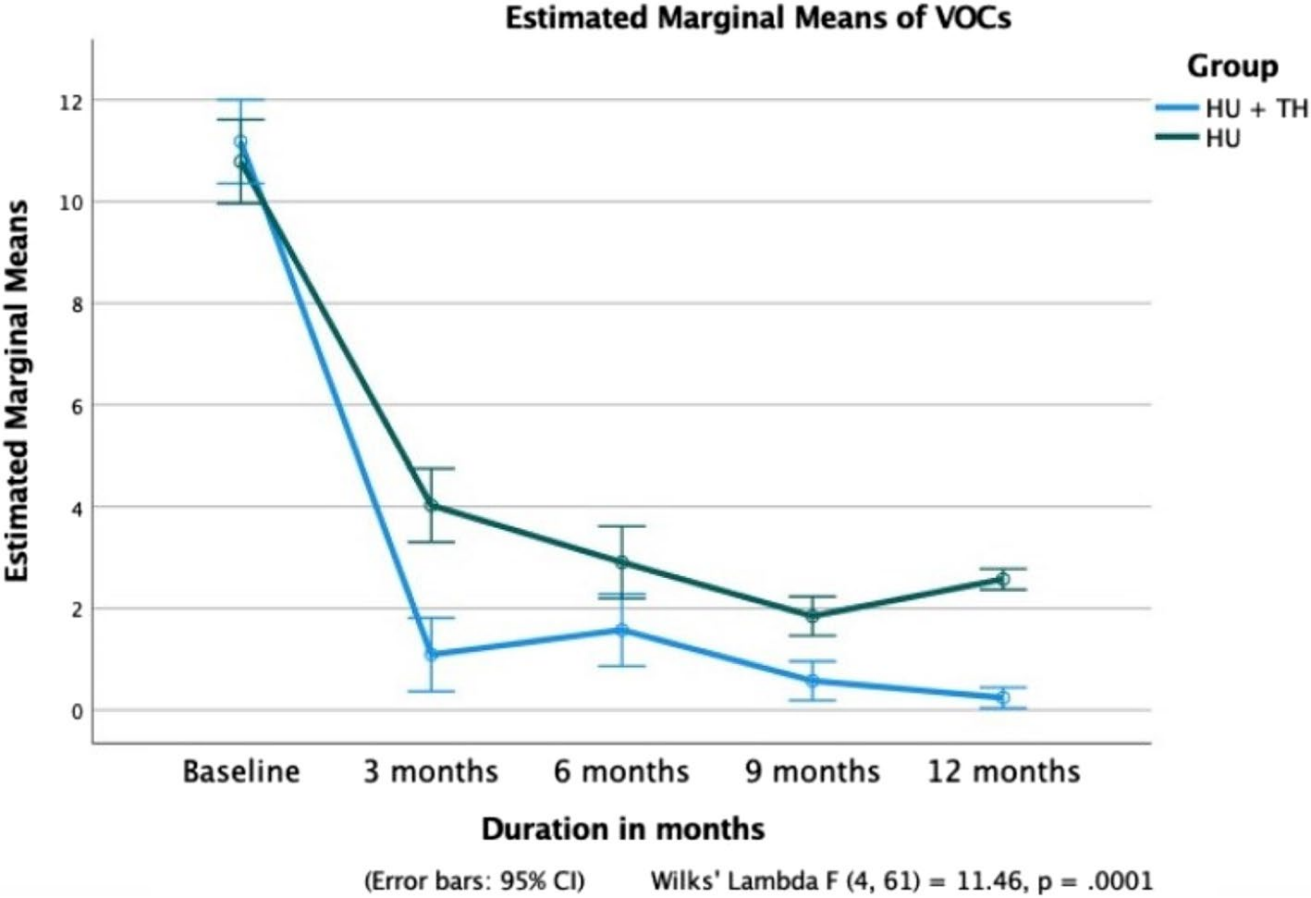
Comparison of VOC episodes between two groups of patients across 12 months

At 12 months, the mean Hb level in Group A patients increased significantly from 8.2 ± 1.8 g/dL to 11.8 ± 1.2 g/dL (*p* < 0.0001), while no significant improvement was observed in Group B (*p* = 0.726) (Fig. 2). A significant reduction in mean HbS% was noted in Group A, decreasing from 72.5 ± 5.5% to 64.5 ± 5.4% (*p* < 0.0001). In contrast, Group B showed a smaller yet significant decline from 73.3 ± 7.7% to 69.9 ± 6.2% (Fig. 4). HbF levels in Group A increased progressively from 18.9 ± 5.1% to 28.4 ± 5.6% (*p* < 0.0001), while no statistically significant increase was observed in Group B (*p* = 0.115) (Fig. 5). Additionally, patients in Group A required significantly fewer PRBC transfusions over 12 months, with a mean of 3.61 ± 2.19 transfusions (Fig. 6).

Table 2 describes the changes in blood parameters across the timeline at 3, 6, 9, and 12 months of treatment compared with those at baseline. At 12 months, the mean Hb level in patients in Group A increased steadily from 8.2 ± 1.8 g/dL to 11.8 ± 1.2 g/dL (*p* < 0.0001), while in Group B, no significant improvement was observed (*p* = 0.726). A steady decrease in mean HbS% from the baseline values of 72.5 ± 5.5 to 64.5 ± 5.4 in 12 months in Group A (*p* < 0.0001) was seen. Similarly, in patients of Group B, a significant reduction in HbS% was observed, albeit to a lesser extent (73.3 ± 7.7 to 69.9 ± 6.2). After treatment, Group A patients’ HbF values increased gradually, from 18.9 ± 5.1 to 28.4 ± 5.6 (*p* < 0.0001) in 12 months. Group B patients did not show a statistically significant increase in HbF values (*p* < 0.115).

**Table 2:** Comparison of clinical and hematological parameters at baseline and over three, six, nine and twelve months

**Variables**	**Follow-ups**					***p* value (Paired Sample t-test)**			
	**Mean ± SD**					**Baseline vs. follow-up months**			
	**Baseline**	**3 months**	**6 months**	**9 months**	**12 months**	**3 months**	**6 months**	**9 months**	**12 months**
**Group A**									
Total PRBC transfusions	3.61 ± 2.19								
Total VOCs	3.48 ± 2.81								
PRBC Transfusions	5.3 ± 1.0	1.8 ± 0.9	1.6 ± 1.6	0.6 ± 0.9	0.2 ± 0.5	0.000	0.000	0.000	0.000
VOCs	11.2 ± 2.4	1.1 ± 1.5	1.1 ± 1.3	0.2 ± 0.6	0.5 ± 0.6	0.000	0.000	0.000	0.000
Hb (in gm/dl)	8.2 ± 1.8	10.4 ± 1.8	11.1 ± 1.6	11.6 ± 1.4	11.8 ± 1.2	0.000	0.000	0.000	0.000
HbS %	72.5 ± 5.5	70.0 ± 5.3	68.6 ± 5.2	66.6 ± 5.3	64.5 ± 5.4	0.000	0.000	0.000	0.000
HbF %	18.9 ± 5.1	23.5 ± 5.1	24.6 ± 5.3	26.1 ± 5.3	28.4 ± 5.6	0.000	0.000	0.000	0.000
Retic count in %	3.3 ± 1.3	3.8 ± 1.1	4.2 ± 1.4	3.8 ± 1.2	3.6 ± 1.1	0.000	0.000	0.042	0.263
MCV (in fl)	83.3 ± 4.4	88.8 ± 5.4	89.6 ± 4.9	91.6 ± 7.0	94.1 ± 6.4	0.000	0.000	0.000	0.000
Creatinine (in mg/dl)	1.7 ± 0.7	1.2 ± 0.8	1.2 ± 0.8	1.0 ± 0.6	1.0 ± 0.7	0.000	0.008	0.000	0.000
Total Bilirubin (in mg/dl)	3.0 ± 1.7	2.4 ± 1.2	2.4 ± 1.0	2.5 ± 1.0	2.5 ± 1.0	0.012	0.028	0.113	0.102
Direct Bilirubin (in mg/dl)	1.2 ± 1.1	0.8 ± 0.4	0.8 ± 0.5	0.7 ± 0.4	0.7 ± 0.6	0.080	0.092	0.025	0.061
Numeric Rating Scale	9.2 ± 0.8	7.1 ± 0.9	4.8 ± 0.8	2.8 ± 0.7	0.4 ± 0.5	0.000	0.000	0.000	0.000
**Group B**									
Total PRBC transfusions	13.27 ± 3.70								
Total VOCs	11.36 ± 4.20								
PRBC Transfusions	5.0 ± 1.6	4.5 ± 1.8	2.9 ± 2.4	2.7 ± 1.3	2.6 ± 1.2	0.095	0.000	0.000	0.000
VOCs	10.8 ± 2.3	4.0 ± 2.6	3.5 ± 1.8	2.8 ± 1.5	2.6 ± 0.7	0.000	0.000	0.000	0.000
Hb (in gm/dl)	9.4 ± 1.1	9.5 ± 1.4	9.7 ± 1.1	9.7 ± 0.8	9.7 ± 1.2	0.756	0.352	0.351	0.429
HbS %	73.3 ± 7.7	71.6 ± 11.3	69.9 ± 10.3	69.7 ± 9.3	69.9 ± 6.2	0.303	0.004	0.004	0.005
HbF %	15.6 ± 6.7	15.0 ± 5.1	16.0 ± 5.0	17.5 ± 5.9	17.1 ± 4.1	0.621	0.694	0.137	0.226
Retic count in %	5.4 ± 2.3	4.0 ± 1.1	3.6 ± 1.0	3.2 ± 0.6	2.7 ± 0.5	0.000	0.000	0.000	0.000
MCV (in fl)	81.6 ± 5.5	83.9 ± 6.6	84.9 ± 5.1	88.2 ± 8.3	88.4 ± 10.9	0.091	0.024	0.001	0.003
Creatinine (in mg/dl)	2.0 ± 1.0	1.5 ± 0.9	1.5 ± 1.2	0.8 ± 0.5	0.9 ± 0.7	0.014	0.088	0.000	0.000
Total Bilirubin (in mg/dl)	5.1 ± 1.6	4.0 ± 3.2	4.5 ± 1.9	3.0 ± 1.2	2.5 ± 1.7	0.105	0.216	0.000	0.000
Direct Bilirubin (in mg/dl)	0.7 ± 0.7	1.3 ± 1.5	1.4 ± 1.2	0.9 ± 0.5	0.9 ± 0.7	0.058	0.013	0.147	0.195
Pain Numeric Rating Scale	9.2 ± 0.7	7.7 ± 1.0	5.5 ± 0.7	3.7 ± 1.2	3.2 ± 1.1	0.000	0.000	0.000	0.000

Patients in Group A had significantly fewer episodes of VOCs (3.48 ± 2.81) and PRBC transfusions (3.61 ± 2.19) over 12 months than VOCs (11.36 ± 4.20) and PRBC transfusions (13.27 ± 3.70) in Group B (*p* = 0.0001 and 0.0001, respectively).

Furthermore, we observed that approximately 85% of the patients in Group A were major responders, exhibiting an increase in hemoglobin of ≥ 2 g/dL, compared with only 15% in Group B, as shown in Table 3. The mean white blood cell (WBC) count was 8.5 ± 4.1 × 103/µL at baseline, which increased to 12.1 ± 15.4 × 103/µL at 3 months and subsequently declined to 7.0 ± 2.5 × 103/µL at 12 months. The mean WBC counts at 6, 9, and 12 months were significantly lower than the baseline values (*p* < 0.05). The mean difference in WBC count between baseline and 12 months was -1.5 ± 3.6 in Group A and -2.1 ± 3.8 in Group B (*p* = 0.561).

**Table 3:** Association of Hb difference at (0–12 months) with groups

Hb (0–12 months) group	Group				Total		*p* value
	Group A		Group B				
	No	%	No	%	No	%	
No responder (< 1 g/dl)	2	6.1	19	57.6	21	31.8	*p* = 0.0001
Minor responder (1—2 g/dl)	3	9.1	9	27.3	12	18.2	
Major responders (≥ 2 g/dl)	28	84.8	5	15.2	33	50	
Total	33	100	33	100	66	100	

The safety profile of the drugs was assessed over the treatment period by serial monitoring of renal and liver function tests, and there were no significant differences between the groups (Table 1). Constipation (grade 1) (*n* = 19, 57.6%) and somnolence (grade 1) (*n* = 6, 18.2%) were the considerable side effects in Group A, while nausea (grade 1) (*n* = 11, 33%) was the predominant adverse effect in Group B. Only two patients in Group A (n = 2, 6%) had grade 1 peripheral neuropathy.

## Discussion

Management of SCA poses a significant challenge because of its chronic and often debilitating nature. HU has been established as a cornerstone in SCA treatment by increasing HbF levels, thereby reducing HbS polymerization and associated complications. However, not all patients respond adequately to HU, necessitating the exploration of adjunctive therapies to optimize outcomes [13]. Several studies have demonstrated the effectiveness of HU as an HbF inducer [14–16].

After a 6-month trial on mean therapeutic dose, HU failure is defined as the inability to reduce the frequency and severity of painful episodes or acute chest syndrome [17]. The ability to tolerate moderate HU doses with little myelotoxicity depends on the “bone marrow reserve,” which, if reduced, leads to HU intolerance even at moderate doses [18]. Recently, thalidomide, which is known to induce immunomodulatory and anti-inflammatory effects in combination with HU, has been studied in vitro for SCA with good outcomes [9].

However, to our knowledge, few studies on patients with SCA have compared the effectiveness and safety of this combination therapy (HU + thalidomide). In this quasi-experimental clinical trial, we investigated the combination of thalidomide with HU compared with HU alone in patients with SCA to evaluate whether adding thalidomide to standard HU therapy could offer better clinical benefits than HU alone.

### Efficacy outcomes

Our findings revealed several notable efficacy outcomes in the combination therapy group (Group A). Patients receiving HU and thalidomide showed a significant increase in Hb levels over the study period, with sustained improvement for up to 12 months. This robust increase in Hb level aligns with previous studies in Eβ thalassemia, which supports the hypothesis that thalidomide enhances erythropoiesis and HbF production synergistically with HU [12, 19–23].

In our study, the mean HbF% in intervention Group A (thalidomide + HU) significantly increased from 18.9 ± 5.1% at baseline to 28.4 ± 5.6% at 12 months (*p* = 0.000). The synergistic action of HU and thalidomide combines nitric oxide-donating properties of HU. This action possibly increases the gamma-globin mRNA transcription and HbF expression in erythroid cells using the pharmacophoric properties of the thalidomide molecule caused by reduced levels of important transcriptional repressors of globin gene expression in erythroblasts, such as BCL11A, SOX6, GATA1, KLF1, and LSD1 [24].

Although direct comparisons were not possible because of the lack of similar trials, a few clinical studies have examined an increase in HbF in patients with thalassemia intermedia. After treatment with single-agent thalidomide, the mean HbF level increases, leading to an improvement in anemia [25]. Moreover, progressive HbF increment with thalidomide was seen in patients with E-β-thalassemia [23]. In Group B (HU), the mean HbF level increased from 15.6 ± 6.7% at baseline to 17.1 ± 4.1%, which was not statistically significant (*p* = 0.226).

Our study showed a higher mean decline in blood requirement in patients receiving HU + thalidomide (3.61 ± 2.19 units) than in those receiving HU alone (13.27 ± 3.70 units) over 12 months of follow-up. Similar results have been produced in patients with Eβ thalassemia with the use of thalidomide alone and in patients with SCD with HU [12, 23, 26]. Given the need for fewer blood transfusions, thalidomide can be considered a cost-effective measure for managing SCA.

The reduction in WBC counts observed in Group A suggests a potential immunomodulatory effect of thalidomide, contributing to the overall hematological stabilization. This finding is consistent with previous studies highlighting the role of thalidomide in immune modulation and its potential to mitigate inflammation in SCA [27, 28].

### Safety and tolerability

The intervention did not require any adjustments based on renal or hepatic functions throughout the study period. Furthermore, in Group A, hepatic and renal parameters steadily improved, although the difference was not statistically significant, especially in the case of serum creatinine levels. A decrease in bilirubin levels following HU treatment has been previously documented [17].

Creatinine levels significantly decreased during the follow-up period in the intervention and control groups. However, no difference was observed between the groups, which can only be attributed to HU, as evidenced by previous studies in the absence of supporting literature on the effect of thalidomide on creatinine levels [29].

Safety assessments revealed a manageable side-effect profile in both treatment arms. Constipation, somnolence, and mild peripheral neuropathy were predominantly observed in the thalidomide group, consistent with known adverse effects of the drug [30, 31]. However, none of these side effects necessitated treatment discontinuation or led to severe complications, emphasizing the manageable nature of thalidomide in combination with HU [32–34].

### Clinical implications

Our findings have significant clinical implications for the management of SCA. The combination of HU and thalidomide has shown promising results in terms of improving Hb levels, reducing transfusion requirements, and decreasing the frequency of VOCs. Achieving transfusion independence in Group A underscores the potential of this combination therapy to alleviate the burden of transfusion dependency in patients with SCA.

Furthermore, the substantial decrease in VOCs and associated pain scores in Group A highlights the potential for enhanced quality of life with combination therapy. This reduction in pain episodes is particularly relevant, given the debilitating nature of VOCs and their impact on patient well-being.

Although our study showed beneficial results, it has a few limitations. This single-center experience limits the generalizability of our results to larger SCA-affected populations. Moreover, while the follow-up duration was sufficient to observe short-term outcomes, it may not have adequately captured long-term safety and efficacy profiles. To further validate the safety and efficacy of thalidomide and HU, future studies should focus on large-scale, multicenter trials with extended follow-up periods.

## Conclusion

This study provides preliminary evidence supporting the efficacy and safety of combining thalidomide with HU for SCA treatment. Combination therapy is a promising therapeutic option for patients who do not achieve optimal outcomes with HU alone, highlighting the need for further research to refine and optimize treatment strategies for SCA management.

## Data Availability

No datasets were generated or analysed during the current study.

## Abbreviations

SCD: Sickle cell disease
HbS: Sickle hemoglobin
RBCs: Red blood cells
VOCs: Vaso-occlusive crises
SCA: Sickle cell anemia
HU: Hydroxyurea
HbF: Fetal hemoglobin
PRBC: Packed red blood cells
WBC: White blood cell
